# Percutaneous cervical coblation as therapeutic technique in the treatment of algo-dysfunctional pain of discal herniation

**DOI:** 10.1007/s11547-021-01336-w

**Published:** 2021-02-23

**Authors:** Marco Pandolfi, Federica Galli, Anna Borelli, Martina Gurgitano, Alessandro Liguori, Gianpaolo Carrafiello

**Affiliations:** 1 Scuola di Specializzazione in Radiodiagnostica, Università degli Studi di Milano, Via Festa del Perdono 7, 20122 Milan, Italy; 2 Unità Operativa di Radiologia Diagnostica e Interventistica, ASST Santi Paolo e Carlo, Polo Ospedaliero San Paolo, Università degli studi di Milano, Via Antonio di Rudinì 8, 20142 Milan, Italy

**Keywords:** Disc protrusion, Discogenic cervicobrachialgia, Visual analog scale (VAS), Nucleoplasty, Mini-invasive therapy

## Abstract

**Objective:**

To confirm the validity of coblation nucleoplasty in reduction of cervical discogenic nature.

**Study design:**

In a monocentric prospective clinical observational study recruiting 20 patients, treated with percutaneous coblation for cervical discogenic pain in 16 months in our hospital, we have clinically evaluated 18 patients. The pain was scored with the Visual Analogic Scale (VAS) in a pre-procedural questionary, 3/4 monthly follow-up from treatment and, finally, in a long-term follow-up 2 years after procedure.

**Results:**

The mean pre-procedural VAS score was 7.9 ± 1.6 (95%—Confidence Interval 7.198–8.634), while the mean post-procedural score after 3–4 months has been 2.5 ± 3.1 (95%—Confidence Interval 1.089–3.965) and 2.5 ± 2.5 (95%—Confidence Interval 1.367–3.687) after 2 years. Among 18 patients, in the shortly post-treatment follow-up, nine had a complete pain relief, four had a > 50% VAS reduction, two had*a* < 50% VAS reduction, three did not have any variation of VAS; after 2 years, six patients had a total pain resolution, eight had *a* > 50% VAS reduction, two had*a* < 50% VAS reduction, two did not have any benefit. No peri- and post-procedural complication has been observed.

**Conclusions:**

In a spite of a little sample, our results showed coblation as a valid therapeutic option to reduce cervical discogenic pain in medicine-refractory patients, as an alternative or a previous choice before a more invasive surgical treatment.

## Introduction

Neck and upper limb pain are a common problem, which often results in functional disabilities and loss of productivity at work. Its prevalence is estimated at between 20 and 45% in industrialized countries, [1] with a higher incidence in older individuals and women, suggesting a correlation with age and sex [2].

All body structures with a sensory nerve supply may be potential sources of neck pain and include muscles, ligaments, bones, zygapophysial joints and intervertebral discs [3, 4].

Among these, it is thought that the pain induced by the stress of the disc, called “discogenic pain”, is one of the most important causes and its prevalence has been reported between 16 and 20% [5, 6].

In 90% of cervical hernias, conservative treatment is sufficient and consists of rest, orthotic treatment with collar, physical therapy, medical therapy with NSAIDs and corticosteroids. Only hernias that are more refractory to conservative therapy are treated with discectomy surgery [7].

Recently, spinal surgery is shifting towards minimally invasive and lower cost procedures such as nucleoplasty; this technique is a minimally invasive procedure, used since 2000 [8].

Coblation consists in the application of radiofrequency energy using a conductive medium, such as a saline solution, to create a precisely focused plasma. The aim is to dissolve soft tissue using relatively low temperatures (typically 40–60 °C), preserving the integrity of surrounding healthy tissue. Nucleoplasty generates small holes in the nucleus of intervertebral discs by combining coagulation and tissue ablation (Coblation technology) through the tip of the Perc-D Spine Wand, and this leads to a decompression of the herniated disc [9].

Chen et al. proved that the decompression using the Coblation technique decreased the pressure in the disc with an experiment in a cadaveric specimen [10].

The main advantage of the coblation technique used in nucleoplasty, versus surgical discectomy, is that exceptionally precise and targeted removal is possible while minimizing the thermal injury to the surrounding tissue [11].

So far, only a few studies evaluated the validity of coblation, in terms of cervical discogenic pain reduction, conferred by the reduction of the disc core volume, reduction of intradiscal pressure, alteration of the expression of inflammatory agents and interruption of nociceptive nerve endings [12–14].

Therefore, the goal of this study is to clinically evaluate the efficacy, side effects and patient satisfaction with cervical nucleoplasty performed on patients with cervical disc disorders that were unresponsive to conservative treatment.

## Materials and methods

### Patients

In a monocentric, prospective, clinical and observational study recruiting 20 patients, treated with percutaneous coblation for cervical discogenic pain, 18 patients have been clinically evaluated in 16 months (ten men, eight women; average age of 52.45). Two patients refused recruiting in the study.

All patients had previously performed a radiological imaging exam (CT: Computed Tomography or MRI: Magnetic Resonance Imaging) and a specialized orthopaedic or neurological examination, proving the discal source of the pain.

Any necessary ethics committee approval was secured for the study reported.

The main inclusion criterion for the nucleoplasty treatment was discogenic pain without motor and sensory deficits not responsive to at least three months of medical treatment and physiotherapy. CT or MRI images should show a discal protrusion, bulging or a little migrated soft herniations.

Exclusion criteria for the procedure included large discal herniations, severe spinal stenosis due to osteophytosis, presence of muscular or ligamentous pain issues, gait disorders depending on different neurological or orthopaedic pathologies. Before the procedures is necessary to know the entire medical history of the patient and carefully investigate clinical and instrumental data; a radiological consultation was carried out before the procedure, to identify therapeutic strategies, to explain to the patients the procedure and the technical approach, to obtain informed consent and to exclude some risk factors, especially the haemorrhagic one: coagulation balance is necessary 1 or 2 days before procedure in order to avoid, though rare, uncontrolled bleeding problems. Benefits and potential risks must always be discussed between the interventional radiologist and the patient or referral doctor.

### Nucleoplasty technique

Percutaneous disc decompression using coblation technology was performed under local anaesthesia by an experienced interventionist radiologist in angiographic room. Antibiotic prophylaxis therapy was administered prior to procedure. A pillow was placed under patient’s shoulders to maintain neck hyperextension. Vital signs were monitored throughout the procedure.

The correct spinal level to be treated was previously chosen by the results of imaging and clinical evaluation.

**Fig. 1:**
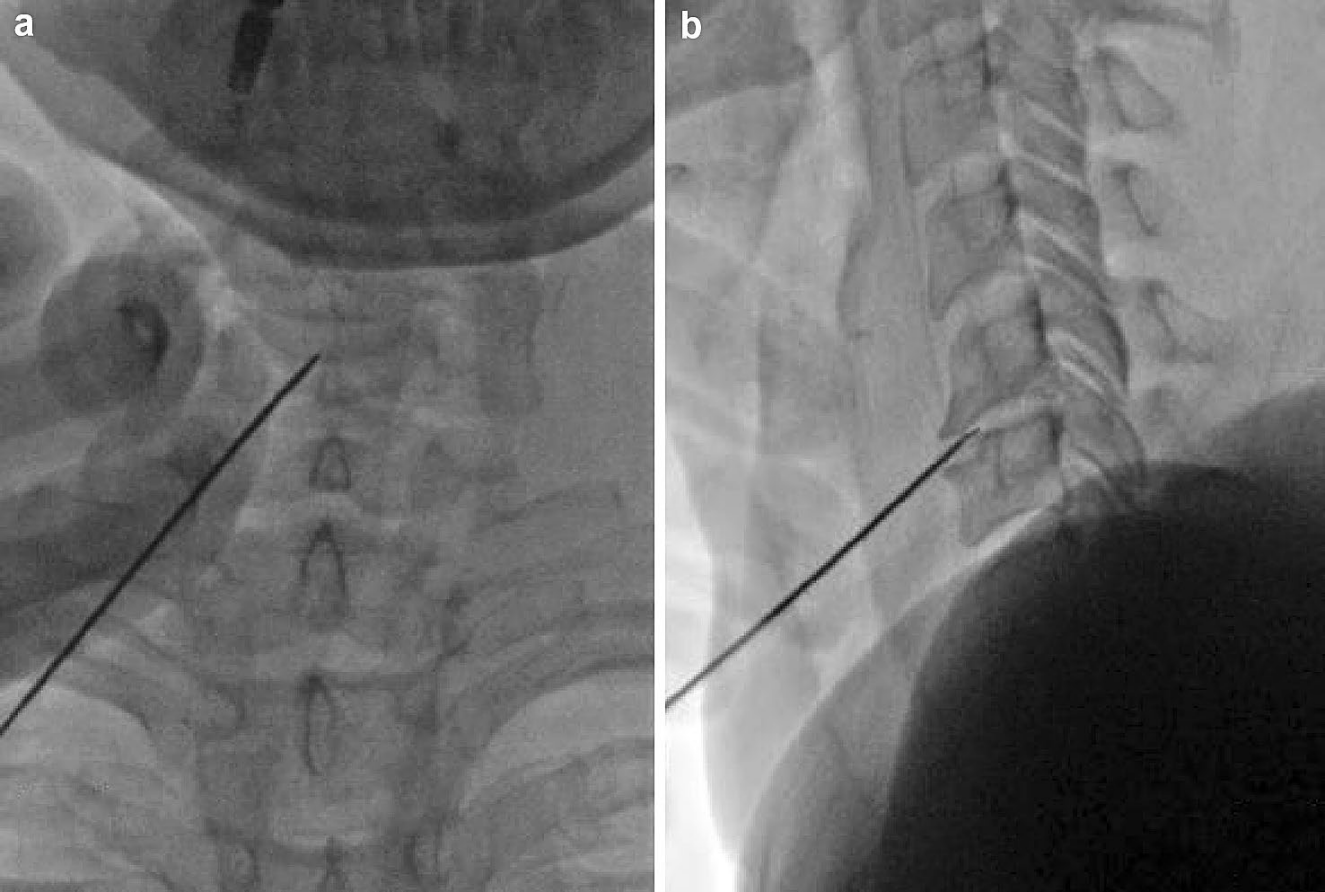
Introduction of Perc-DLE spine wand in a 43 years old woman with C5–C6 discal protrusion. (**a** Antero-Posterior view, **b** Latero-Lateral view)

Before starting the procedure, anterior cervical spine was palpated with fingertips and larynx and trachea were displaced medially, while the carotid artery was displaced laterally. Under fluoroscopic guidance, the puncture angle was confirmed using a lateral–lateral view. To reach the target disc at the juncture of anulus and nucleus the needle was introduced with a single-sided anterior approach. The exact position of the needle tip was confirmed on the anterior–posterior and lateral–lateral views, as shown in Fig. 1. Once the appropriate position of the needle is verified, the stylet is removed, and the Perc-D tissue ablation and coagulation SpineW and radiofrequency probe (ArthroCare, Inc.—Sunnyvale, CA) is placed through a coaxial approach, assuring that the active portion of the wand was placed in the nucleus. After reaching the nucleus, the wand is advanced to the opposite edge of the annulus and repeated movements are made to create more channels within the nucleus. Specifically, each channel is made by advancing the wand in ablation mode for 6–8 s, followed by the retraction in coagulation mode for 10–15 s. To obtain an adequate disc decompression, a total of six channels were created at the twelve, two, four, six, eight, and ten o’clock positions.

The procedure results in tissue removal (via plasma dissociation) able to guarantee both disc decompression and chemical denaturation of sensitive nerve fibres whose growing within the disc nucleus.

We observed patients at least 6 h after procedure and patients were advised to stay in bed for the 1st day following the procedure. If necessary, non-steroidal anti-inflammatory drugs and myorelaxant drugs may be prescribed to the patients but it is an option to be assessed in relation to the patient’s condition. No lifting of weights, bending, or stooping was permitted for 2 weeks following percutaneous disc decompression. Patients were returned to sedentary or light work after two weeks and were provided with home exercise instructions by a qualified physical therapist.

### Pain evaluation

Once the discogenic cause of the pain has been recognized, the pain symptomatology has been evaluated by Visual Analogic Scale (VAS): the patient had compiled it immediately before the procedure and after, respectively, 3/4 months and 2 years from the treatment, when they came back for a clinical follow-up.

VAS scale is a standardized tool to measure pain. Patients assessed the intensity of their subjective pain on a 10 cm long scale, with values from 0 (no pain) to 10 (the greatest pain imaginable) with a space of one centimetre between the individual values [15].

## Results

The results obtained by 18 patients (10 males and 8 females) with discogenic cervicobrachialgia treated with nucleoplasty procedure, using the coblation technique, were analysed.

Four of the 18 patients received treatment at the level of two distinct discs, of which three during the same session and one in two separate moments.

Coblation was therefore performed on a total of 22 intervertebral discs: three at the level of the C3–C4 disc (13.63%), three at the level of the C4–C5 disc (13.63%), in 13 cases for the disc between C5–C6 (59.09%) and finally in another three cases for the disc between C6-C7 (13.63%).

The characteristics of the patients regarding the distribution by age, sex and level of the treated pathology were presented in Table 1.

**Table 1:** Features of patients regarding the distribution by age, sex and level of the treated pathology

Mean age (range)-years	52.45 (33–77)
Gender	
Men (%)	10 (55.55%)
Women (%)	8 (44.45%)
Treatment level	
C3–C4 (%)	3 (13.63%)
C4–C5 (%)	3 (13.63%)
C5–C6 (%)	13 (59.09%)
C6–C7 (%)	3 (13.63%)

The pre-operative baseline mean VAS score was 7.9 ± 1.6 (95% confidence interval 7.198–8.634 with a total range between a minimum of 5 and a maximum of 10), while the mean post-operative score was 2.5 ± 3.1 after 3/4 months (95% confidence interval 1.089–3.965 with a total range between 0 and 9) and 2.5 ± 2.5 (95%- Confidence Interval 1.367–3.687 with a total range between 0 and 9) after 2 years.

**Fig. 2:**
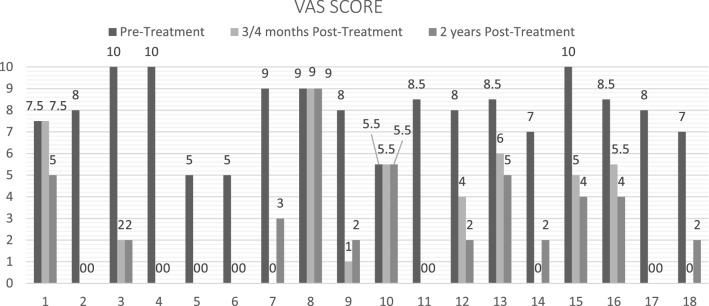
Histogram showing the differences between pre-treatment (black column), post-treatment after 3/4 months (light grey column) and 2 years subsequently to the procedure (dark grey) VAS score in the 18 patients

The variation of the VAS scale in the pre-operative and post-operative phases, divided for each individual patient, is illustrated in Fig. 2.

In the short follow-up of the 18 patients, nine (50%) showed a total remission of symptoms, four (22.22%) a reduction of more than 50%, two (11.11%) a reduction of less than 50%, while in three cases (16.67%) the VAS value was unchanged; in the later follow-up after 2 years, six patients had a complete pain relief (33.33%), eight had* a* > 50% VAS reduction (44.44%), two had*a* < 50% VAS reduction (11.11%), two did not have any change (11.11%).

**Fig. 3:**
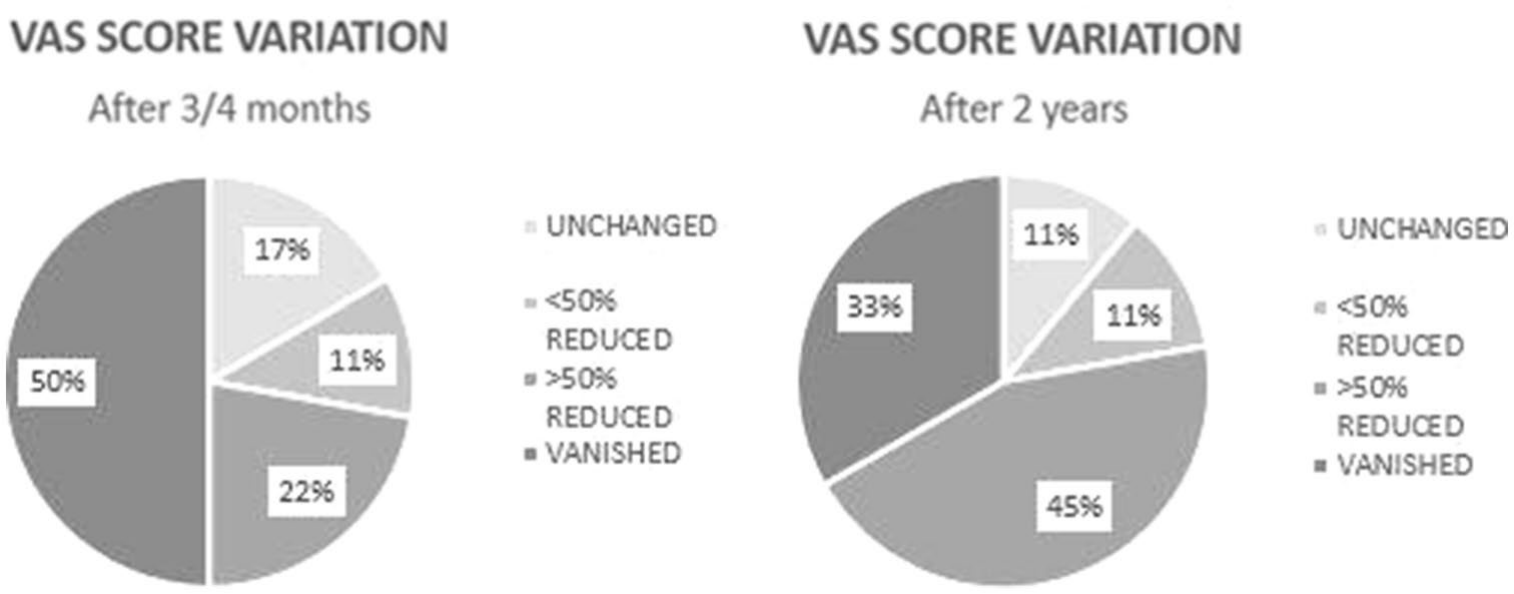
Pie chart showing the variation of the VAS scale in the pre-operative and post-operative phases (after 3/4 months and after 2 years), divided in four classes: total remission of symptoms, reduction of more than 50%, reduction of less than 50% and unchanged

These data are summarized in Fig. 3.

All our patients had failed pain medications prior the procedure: 15/18 had only medical treatment with NSAIDs and corticosteroids, 2/18 had medical treatment with NSAIDs and corticosteroids and physio kinesitherapy and 1/18 had medical treatment with NSAIDs and corticosteroids and spinal injection.

Two years after treatment, we noticed a clear reduction in the use of drugs: 2/18 use NSAIDs just in the acute phases, 1/18 has only occasional sessions of physio kinesitherapy and 2/18 underwent to open discectomy.

There were no severe peri and post-procedural complications, as bleeding, paresthesia and infections. Three patients complained of pain immediately after treatment, while in another two patients a small hematoma was observed at the puncture site; both conditions regressed in about 1 week, without long-term discomfort.

## Discussion

In this study, it was evaluated the efficacy of nucleoplasty that is a minimally invasive technique for the treatment of discogenic neck pain. Already in 2003, Chen et al. demonstrated that the application of RF energy on the disc material results in breakdown of molecular bonds of the tissue into elementary molecules and low molecular gases. The whole procedure results in removal of the disc material and reduction in intradiscal pressure [16].

The etiopathogenesis of the discal stress is a sum of micro-traumatic and degenerative factors of the disc leading to a change in the chemical composition of the nucleus pulposus and the fibrous anulus, resulting in a progressive reduction of the viscoelastic properties [7, 17] for the loss of proteoglycans and water. Because of the lack of nutrients and oxygen, cells are forced to metabolize anaerobically, generating a large amount of lactic acid; it leads to an increase in acidity resulting in further degradation of the intradiscal matrix [18]. Some studies have shown that degenerated cervical discs can produce direct neck pain due to the presence of nerve fibres and terminal nerves located in the peripheral portion of the disc [19, 20].

Disc degeneration and its reduction in height, determines a posterior dislocation in the medullary canal and intervertebral foramen, inducing a potential radicular conflict; also, arthrosis-related damage like osteophytes contributes to increase extrinsic pressure on the nerve root as well as on the spinal cord, leading to both sensory and motor deficits [7, 17].

In clinical practice, the terms "soft hernia" and "hard hernia" are commonly used to describe and distinguish different disease that may affect cervical spinal tract; soft hernia is used to define discal material recently herniated while hard hernia refers to earlier discal material, partly dehydrated and replaced by calcified tissue. No significant clinical differences are found, even if soft hernias may have a more acute onset [21].

In most cases, the hernia is placed in a posterolateral position and conflicts with the root emergency, giving a radicular discogenic pain typified by painful symptomatology radiated to the upper limb (generally only one), disabling, often present even during the night, accompanied by disorders of the tactile sensitivity and pain due to motor deficit and modification of the osteo-tendon reflexes [22].

In the most severe cases and in a smaller percentage of patients, the hernia may instead take a more central position and cause direct compression on the anterior portion of the spinal cord. We will have a different symptomatology, involving both the upper and lower limbs: the so-called cervical myelopathy modifying osteo-tendon reflexes, with absence or reduced grip strength at the level of compression and hyper-reflex below it; appearance of pathological reflexes; hypo-amyotrophy of muscle groups innervated by fibres from the anterior horns cells of the damaged spinal cord; fasciculations and fibrillations; sensitivity disorders that travel along the posterior cords (most compromised in the stenosis on a degenerative basis) [4].

So, the symptoms can lead to a presumed diagnosis about the discal level involved by pathology, as describes by Stephen A. Grubb et al. in 2000: they analysed the results of 12 years of study and described the various pain patterns that were triggered by the stimulation, by cervical discography, of each cervical disc from C2 to C7 [23].

First, in our study, we had to consider the selection of a suitable patient in proceeding with cervical nucleoplasty. Nardi et al. [12] argued that patient who can be a candidate for cervical nucleoplasty must have contained herniation or focal bulging proven on MRI, but Bonaldi et al. [24] reported that the cervical nucleoplasty was performed in the patients who had a bulging, protruding or soft-extruded disc which was not sequestrated or migrated determined by MRI or CT studies.

In our study, the selected patients were suffering for a discogenic pain, not associated to significant motor or sensitive deficit, refractory to upto 3 months conventional therapy, with a discal protrusion or a little migrated soft herniations on CT or MRI images. We preferred to treat hard hernias using a mechanical disc decompression, disintegrating the calcified intradiscal components.

Exclusion criteria included large discal herniations, spinal stenosis with osteophytosis and an underlying neurological or orthopaedic disease.

Secondarily, we can notice that in our study the most involved disc has been C5–C6, as in the analysis of Suzuki et al. [25]. This huge study shows that the patient group with 1-level degeneration, C5/6 was the most common degenerated level (51.2%), followed by C4/5 (19.8%) and C6/7 (16.7%). It could suggest the most common site of stress for cervical intervertebral discs when the pain has a discogenic origin.

The main consideration of our study is about the significant reduction of algo-dysfunctional cervical pain due to discogenic degeneration, with a significant lifestyle improvement of treated patients.

Regarding the mean VAS score, we noticed a substantial reduction from a presurgical score of 7.9 to a post-surgical value of 2.5 after 3–4 months and after 2 years from coblation. Our results are according to values of Birnbaum et all in 2009 [9] however a little lower; indeed, they obtained a VAS pain reduction from 8.8 presurgical to 2.0 after 3 months from the treatment, even if they showed a small deterioration to 2.3 after 24 months.

Moreover, we detected in the first 3/4 months after procedure a total pain resolution in 50% of patients, a more than 50% reduction in 22.22%, a less than 50% decrease in 11.11%, while in 16.67% we did not obtained any variation of symptoms. We also noticed a slight worsening of results after 2 years, six patients had a complete resolution of pain (33.33%), eight had *a* > 50% VAS reduction (44.44%), two *a* < 50% VAS reduction (11.11%), two didn’t have any change (11.11%).

Even in this case, our results are according to a 2007 study of Calisaneller et al. [26], 93.1% of patients had an initial decrease in pain after three months, even if only 48.28% had a reduction more than 50%.

Regarding the limited long-term decline in results, it is possible that coblation may cause some degenerative effects on the treated levels which may result in an increased discogenic pain.

One important result of our study was to notice a clear reduction using drug therapies: before treatment, all 18 patients had medical treatment with NSAIDs and corticosteroids, 2/18 had also physio kinesitherapy and 1/18 moreover had spinal injection.

After treatment 13/18 report no longer using drugs, 2/18 use NSAIDs just in the acute phases, 1/18 has only occasional sessions of physio kinesitherapy but 2/18 underwent to open discectomy.

**Fig. 4:**
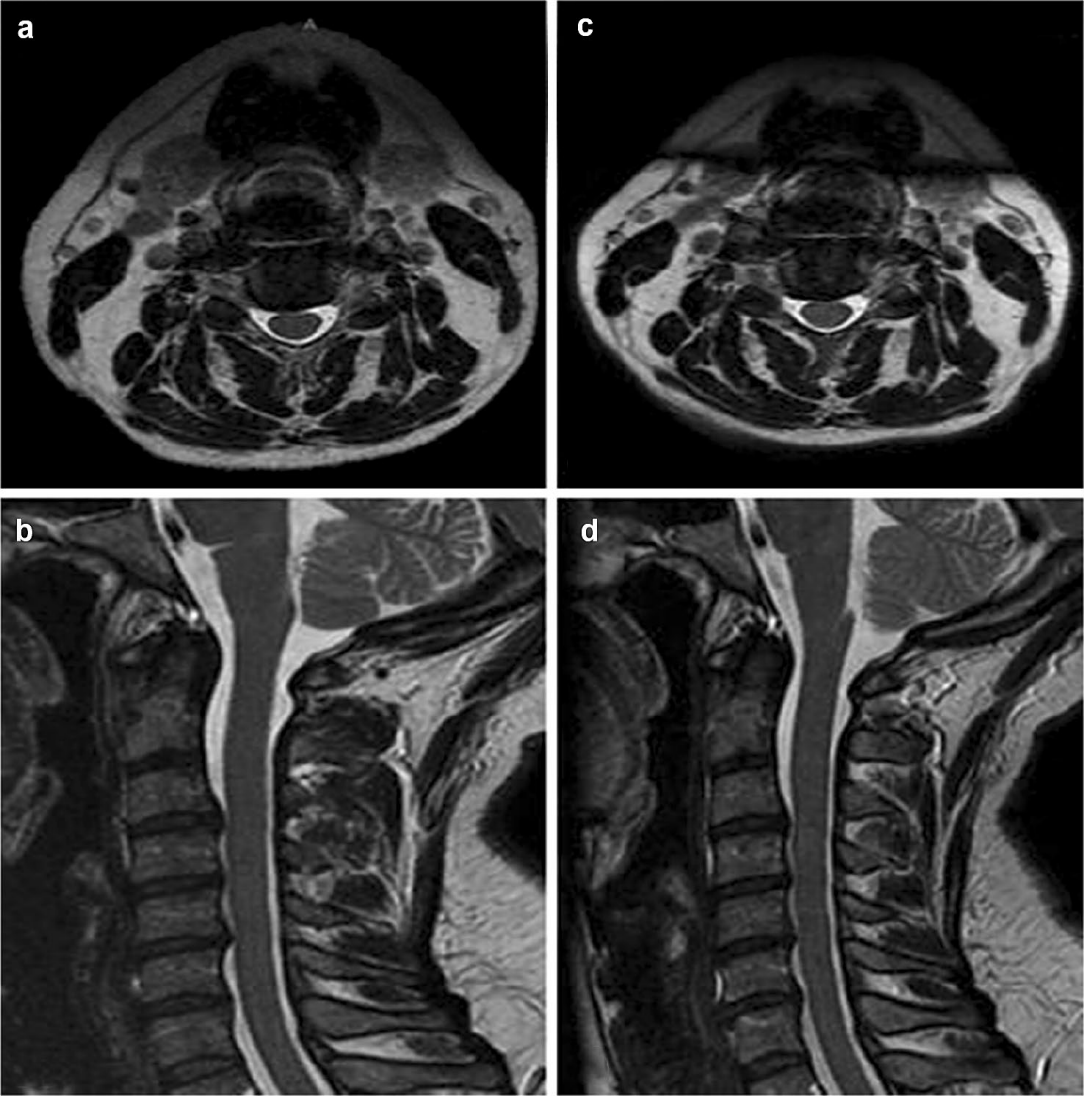
Axial and sagittal T2 MR images pre-treatment (**a**, **b**) and post-treatment (**c**, **d**) in a 46 female patient with pain reduction more than 50%: in this case, we had not significant MRI changes of the discal protrusion, despite of clinical improvement

Just 3 patients had MRI study after treatment: an intervertebral disc volumetric reduction was seen but no substantial signal changes are registered if compared with the baseline survey. We decided not to extend MRI evaluation protocol to all Patients, in order to emphasize post-procedural clinical changes and drugs taken. In the Fig. 4 we report the MR images pre- and post-treatment of one patient with pain reduction more than 50%.

These results of cervical nucleoplasty appear to be much better than the results of lumbar nucleoplasty. However, the reasons that the nucleoplasty treatment may be more effective at the cervical level than at the lumbar level are not clear. One possible explanation could be anatomic: the cervical nerve root is confined to a relatively smaller space than its lumbar counterpart, so the cervical nerve root responds more sensitively to even a minute reduction. For this reason, even if the pressure of the disc is reduced slightly, the decompression on the nerve root and reduction in clinical symptoms can be easily obtained. Another reason could be the topography of the lesion and direction from which it is approached for treatment. In lumbar nucleoplasty, we use a posterolateral approach from the lesion. But in cervical nucleoplasty, we use antero-lateral approach to the disc, and so, the SpineW and TM could be accurately positioned in the lesion site posteriorly located. In other words, since symptomatic herniation is directed posterior, cervical nucleoplasty can effectively approach the lesion site because it uses the anterior approach [11, 13, 24, 27, 28].

On the other hand, there's a study of our group [18] that obtained excellent results even in treatment of lumbar herniations.

In the end, we can argue about side effects or complications of this procedure.

Few side effects concerning the cervical nucleoplasty have been reported and no serious side effects have been known; potential serious complications of nucleoplasty are neurovascular injury to adjacent structures or post-operative spondylodiscitis.

Side effects reported in literature included cases of infectious discitis, temporary side effects related to local anaesthetic (bradycardia, Horner’s syndrome, hoarseness, etc.) and retrosternal and retropharyngeal pain, but they responded well to conservative treatment [24, 29].

We too did not encounter any serious complications in our study, moreover in none of our patients a pain raise was noticed also. Three patients complained pain soon after the procedure, while other two developed a little hematoma in the puncture site.

Surgical technique has been widely accepted as the standard treatment for more than five decades. However, this procedure is associated with a small risk of serious complications such as perforation of the oesophagus, injuries to the carotid or vertebral arteries and severe neurological complications. However, the results of surgery are not always satisfactory, and the overall outcome may be similar to conservative treatment [30].

Many minimally invasive spinal surgical (MISS) alternatives using microscopy, laser technology, endoscopy, and video and image guidance systems have been proposed in the past decades to reduce the morbidity of surgical procedure, to minimize the risk of iatrogenic injury resulting from open discectomy and, consequently, to decrease health care costs [31].

In this perspective, even if actual literature data regarding coblation technique are poor and incomplete, some studies exalted its results so that coblation can be considered as intermediate option between a conservative treatment and a surgical one, especially in patients with degenerated and protruded disc, not responsive to a standard medical-rehabilitative therapy patients and not-surgical-eligible patients.

Coblation benefit is due to its ability to remove part of nucleus pulposus with minimal invasiveness, through its volume reduction, intradiscal pression reduction and biochemical status change [32–34].

Among percutaneous treatments, great results have been reached in chemiodiscolysis performed by administration of intradiscal oxygen–ozone (O2–O3) evaluating the Oswestry Disability Index (ODI) and pain intensity (0–5) scale [35]; moreover a recent study explain how MRI changes on T2 mapping could be useful to predict disc shrinkage and the clinical response to CT-guided O2–O3 injection [36].

However, there are some limits in all these studies, due to poor literature, few sample sizes, absence of control group, short follow-up, limited clinical indications, poor imaging-clinical correlation.

In the future, it will be important to increase sample sizes, to create clinical randomized studies and long-term follow-up, to define inclusion/exclusion criteria, to promote multidisciplinary approaches, to improve executive technique.

According to our experience, disc decompression using the Coblation technique has proved to be one of the best MISS, but it is essential to strictly adhere to his indications and to choose the best therapeutic approach for each patient.

## Conclusion

The results of our study have shown that coblation is a satisfactory, safe and minimally invasive procedure, and is a valid therapeutic alternative to surgical discectomy in reducing allergic symptoms for the treatment of cervicobrachialgia of a discogenic nature, with a more than acceptable response in the short and medium–long terms.

Small cohorts and the absence of a control group, although perhaps unfeasible, are the weak points of this study; therefore, further randomized placebo-controlled studies and large recruitment are necessary to clarify the effects of nucleoplasty on discogenic neck pain.

## Availability of data and material

Datasets analysed during the current study are available from the corresponding author on reasonable request.

## Data Availability

Not applicable.
